# A Systematic Review and Comprehensive Critical Analysis Examining the Use of Prednisolone for the Treatment of Mild to Moderate Croup

**DOI:** 10.2174/1874434601711010241

**Published:** 2017-11-30

**Authors:** Anna Catherine Elliott, Graham R. Williamson

**Affiliations:** 1 Meneage Street Surgery, 100 Meneage Street, Helston, Cornwall TR17, 8RF, UK, 01326 555288; 2 Adult Nursing, School of Nursing and Midwifery, Plymouth University, Drake Circus, Plymouth, PL4 8AA. UK, 07976761858

**Keywords:** Systematic review, Croup, Prednisolone, Dexamethasone, QualSyst, Corticosteroids

## Abstract

**Background:**

Many randomised control trials and systematic reviews have examined the benefits of glucocorticoids for the treatment of croup in children, but they have reported mainly on dexamethasone as an oral treatment for croup. No systematic reviews have examined prednisolone alone.

**Aim:**

To determine in a systematic review of the literature whether a single dose of oral prednisolone is as effective as a single dose of dexamethasone for reducing croup symptoms in children.

**Search Strategy:**

A detailed search was conducted on the following databases: CINAHL, MEDLINE EBSCO, MEDLINE, OVID, PubMed, The Cochrane Library, ProQuest, EMBASE, JBI, Sum search, and OpenGrey. Study authors were contacted.

**Selection Criteria:**

Randomised Controlled Trials, clinical trials or chart reviews which examined children with croup who were treated with prednisolone alone, or when prednisolone was compared to a dexamethasone treatment and the effectiveness of the intervention was objectively measured using croup scores and re-attendance as primary outcomes.

**Data Collection and Analysis:**

Following PRISMA guidelines for systematic reviews, relevant studies were identified. Scores were graded agreed by two independent reviewers using QualSyst.

**Main Results:**

Four studies met the inclusion criteria, but were too heterogeneous to combine in statistical meta-analysis. The result suggests that although prednisolone appears as effective as dexamethasone when first given, it is less so for preventing re-presentation. Trial sample sizes were small, making firm conclusions difficult, however, a second dose of prednisolone the following day may be useful. More research including cost-benefit analysis is needed to examine the efficacy of prednisolone compared to dexamethasone.

## 1 BACKGROUND

### 1.1 Prevalence of Croup

Croup or acute laryngotracheobronchitis is a common illness, which is most prevalent in young children between the ages of 6 and 36 months, and has a large impact on health services. Croup is a common reason for accessing out-of-hours care [1] and is a frequent cause behind hospital admissions of children between 6 months and 3 years [2]. Admission rates of children assessed in outpatient settings range from 1.5% to 31% of the cases seen, depending on hospital admission policies, the severity of the disease, and the social and other characteristics of the population being assessed [3].

Usually appearing as a self-limiting illness, croup often causes parental anxiety, and imposes a large burden on healthcare systems [3]. However, most symptoms of mild croup usually resolve within 48 hours. In some cases, severe upper-airway obstruction can lead to respiratory failure and arrest, but such cases are rare [4]. Most children are managed through primary care, with less than 5% requiring hospitalisation, among which 1-3% require ventilator support in an intensive care setting [5].

The economic burden imposed by croup has been demonstrated in a study by Rosychuk *et al*, [6] which reported that there were 27,355 emergency admissions at accident and emergency settings in the province of Alberta, Canada, for episodes of croup over a six-year period. The incidence of croup among children is reported at three children per one hundred children in the United States in a typical year, with as many as 6% of those children requiring hospitalisation [6]. Figures are similar in Australia, with croup affecting 3% of children under 6 years of age in a typical year [7]. One retrospective Belgian study found that 16% of children aged 5-8 years had suffered from croup at least once and 5% of those children had experienced recurrent croup (at least 3 episodes) [8].

Respiratory tract infections such as croup in children are one of the most common reasons for parents consulting health professionals, with viral croup being the most common form of airway obstruction in children between six months to six years [9]. One long-term prospective cohort study suggested that croup occurred most commonly in children aged between 6 months and 3 years, but can also occur in children as young as 3 months and as old as 12-15 years [10].

### 1.2 Aetiology of Croup

Croup is more prevalent during autumn and early winter with major peaks coinciding with Para-influenza activity often observed in October [11]. The following are the most common viral causes for croup: Para influenza type 1 and 2, Influenza A, adenovirus, rhinovirus, respiratory syncytial virus and, mycoplasma pneumonia [12]. The Para influenza virus type 1 accounts for approximately half of all cases throughout the winter [13]. These common viruses can affect the narrowest part of the airway called the subglottic region, and even small amounts of swelling or oedema can significantly increase the difficulty involved in breathing in young children [14]. As oedema of the proximal airway epithelium progresses, inspiratory stridor and signs of breathing difficulties supervene [15]. This is due to the small children having a very narrow larynx such that even a small decrease in airway radius can cause a large decrease in airflow, leading to croup symptoms. Commonly between the ages of 1 and 3 because as children become older, their breathing tube becomes firmer and wider and so the incidence of croup reduces [1].

Several other factors may make a child more likely to suffer with croup. This may be due to the pre-existing narrowing of the upper airway, subglottic stenosis (congenital or secondary to prolonged neonatal ventilation) or Downs syndrome [16]. Furthermore, Alshehr **et al.**, [17] suggests that there is increasing evidence that an immunological component for acute croup coincides with high titres of both Para influenza virus and specific immunoglobulin E in the children’s nasal secretions. This may explain why croup will often affect atopic children who may already suffer with asthma or eczema.

### 1.3 Corticosteroids in The Treatment of Croup

Oral corticosteroids act on the subglottic oedema and obstruction by decreasing capillary permeability and suppressing localised inflammation [18]. The exact mechanism of the administration of steroids in croup is not fully understood [13] but it is known that glucocorticoids also have vaso-constrictive effects that may contribute to their clinical actions by resulting in reduced airway oedema, less micro-vascular leakage, and reduced airway mucus production [19]. When used as croup treatments, glucocorticoids reduce the inflammation in the airway obstruction as quickly as one hour after administration [20]. A reduction in these inflammatory properties leads to a decrease in the difficulty of breathing for the child.

Prednisolone or dexamethasone may be given orally or intramuscularly, both of which have superior efficacy to placebo but oral preparations of corticosteroids are the preferred mode of administration in most paediatric emergency departments because they are inexpensive, easy to administer, readily available, and result in measurable improvements [21]. Systemic corticosteroids (dexamethasone, prednisolone) are the treatment of choice because benefits can be seen in patients with all levels of croup severity [22].

The half-life of dexamethasone is approximately double of that of prednisolone, and estimates range from oral dexamethasone having an effective half-life of 48 hours, compared with the 24 hours’ half-life of prednisolone to prednisolone’s relatively short-acting half-life of 12-36 hours, thereby requiring daily dosages [23, 24]. In comparison, prednisolone is up to six times less potent than dexamethasone with hypothalamic-pituitary-adrenal axis suppression in prednisolone lasting 1.5 days against 2.5 days in case of dexamethasone [20]. Extensive evidence in the form of primary studies [14, 25-28,] shows that the administration of steroids in a single dose of corticosteroid lessens the risk of a child needing hospital admission or re-presenting for further medical care. Meta-analysis of 24 randomised controlled trials [25] reviewed nine methodically satisfactory trials, five favourable and four unfavourable, involving steroids in the treatment of croup, which supported the use of steroids for children who were ill enough to be hospitalised. Among the included studies, dexamethasone, budesonide, and prednisolone were all included and the results were amalgamated, which made it not possible to identify each arm of the group, instead they were examined as a whole cohort. This meta-analysis concluded that glucocorticoids were effective at improving symptoms within six hours, for up to 12 hours [25]. A further meta-analysis [28] reports main outcomes, clinical improvement, and croup scores of the children at 12 and 24 hours post treatment and the incidence of endotracheal intubation, and provided a reliable estimate of the impact of steroid therapy on the morbidity associated with croup at that time. Reported data from the ten clinical trials involving children with severe croup showed that corticosteroid treatment decreased endotracheal intubation fivefold. Children with mild croup only require reassurance, suggesting that at that time there was no evidence that steroids have a place in management in this group, although a single dose of prednisolone is probably appropriate for children with ‘stridor at rest’, but no recession [29].

Evidence clearly supports the use of oral, parenteral, or nebulised corticosteroids for children admitted with croup [3, 14] and it has been demonstrated that the glucocorticoid treatment of croup has consistently led to improvements in symptoms so much so that they infer that studies involving just dexamethasone are unwarranted as there is so much evidence that it works, but there is a lack of direct comparison studies of dexamethasone *versus* prednisolone, with very little literature available [30]. However, concerns exist about using oral steroids to treat croup, as these types of drugs with their long serum half-lives could have sustained effects on multiple systems, and may reduce the immune function [17]. One study [31] reports that four or more courses of oral prednisolone during childhood may have adverse effects, including an increased fracture risk, although there are no relevant comparison studies. It appears that the risks of administering single-dose corticosteroids are very low, but should be considered in children with diabetes mellitus, children exposed to varicella virus, children at risk of bacterial super infection (*i.e.*, those who are immunocompromised), or have gastrointestinal bleeding [32]. The small benefits of steroids for mild croup have been discussed as not worth the risk of serious adverse effects which can be seen in as many as eight children per thousand who are treated with steroids [33]. A Cochrane review [34] disputed such safety concerns, providing comprehensive safety data on the use of steroids in 2,214 children with acute respiratory conditions, including croup and other respiratory problems such as asthma, concluding that the results showed no difference between children receiving steroids *versus* a placebo 1.5% V 1.8% (gastrointestinal bleeding and or abdominal pain in the steroid group *versus* the placebo group). Gastrointestinal bleeding would be unlikely in otherwise healthy children [12].

## 2 RATIONALE FOR THIS SYSTEMATIC REVIEW

In the United Kingdom, there is currently a nationwide difficulty in obtaining dexamethasone syrup for the treatment of croup in children, which has led to the default prescription of the only other alternative, prednisolone. In some areas in the UK, clinicians have been unable to obtain dexamethasone syrup for some time, and dosing regimens vary between emergency, primary and secondary care settings. Many children with croup are being treated with a one-off dose of prednisolone, although a two or three-day treatment at varying doses has also been noted, with the prescribing of prednisolone appearing to be used in a similar way to treating exacerbations of asthma in children, with up to a three-day course; however, there is confusion in the available literature about what dose of prednisolone to give and for how long. It is not clear what the most therapeutically effective range is and this lack of clarity drives the need for this review. Commercially, at the time of writing, oral dexamethasone was only available locally as a tablet, which makes it unsuitable for young children [5]. Hospital pharmacists can make it up in the form of a dexamethasone elixir, but that is expensive and not available in general practice [35]. Prednisolone has pharmacokinetic properties similar to dexamethasone, and has the significant advantage in that it is commercially available in a liquid preparation. Prednisolone can be prescribed as a soluble tablet; which parents can crush into a little juice or water to give to their children.

In Australia, where a majority of the major croup studies have taken place, oral dexamethasone suspension is only available in hospitals and not at commercial pharmacies, thus necessitating the use of prednisolone in primary care settings [16]. Liquid dexamethasone preparations are also not available in many counties, including Germany and most other European countries [36].

## 3 MATERIALS AND METHODS

The purpose of systematic reviews is to determine effectiveness and involve the comparison of two or more interventions [37]. Systematic reviews of other types of evidence can facilitate decision-making in areas where randomised controlled trials (RCTs) have not been performed or are not appropriate [38]. They are considered the best way to synthesise the findings of several studies, all investigated in the same way [39] noting that systematic reviews have become the gold standard for evidence-based decision-making, and provide building blocks for clinical practice guidelines [40]. Systematic reviews are essential to summarise evidence relating to efficacy and safety of healthcare interventions. With their synthesis of a large body of evidence they aid policy makers and professionals to keep up-to-date with advances [39].

Systemic reviews and meta-analysis provide the highest level of evidence. However, poor reporting may reduce their utility. The PRISMA statement was developed to help authors report their systematic reviews adequately [41] and has been incorporated into this systematic review. There are several approaches to this process but all follow similar steps, which are identifying the problem, finding the research, and determining the level of evidence [42]. The PRISMA statement aims to improve reporting focussing on systematic reviews of RCTs and systematic reviews that need to adhere to rigorous methodology to produce clear and unbiased results [43].

Centre for Reviews and Disseminations (CRD) [44] recommend that before undertaking a systematic review, researchers should check whether there are existent or on-going reviews on their area of interest, and whether a new review is justified, beginning the process by searching the Database of Abstracts of Reviews of Effects (DARE). To date, there are no other systematic reviews that specifically look at the issues of whether prednisolone is as effective as dexamethasone for this very common childhood complaint, which makes this work important. Yet it is acknowledged that there are several systematic reviews of all treatments for croup, which include prednisolones usage, and some of which compare prednisolone to dexamethasone [14, 20, 25-28]. However, no new systematic reviews have been conducted since 2011.

### 3.1 Review Aims

To investigate the comparative effectiveness at reducing croup in children under 12 years of oral dexamethasone and prednisolone, and to examine the optimum dose of prednisolone to prevent relapsing symptoms and re-admission.

### 3.2 PICO

Using PICO helps structure a search, and should include the main terms relating to the research question [45]. PICO is one of the tools that can assist in formulating research questions [44], and a clear concise question will make it easier to generate the best available evidence [46]. The PICO developed for this systematic review is shown in (Table **1**).

### 3.3 Population

The population for this study is children who have croup. The area of interest is the treatment or therapy of children suffering with croup. The age range for this review is set at 3 months-12 years [1] as this is the most typical age bracket during which children are affected. Gender is not a variable in this study, although it is noted that croup typically affects more boys than girls.

### 3.4 Intervention

The intervention will examine the use of prednisolone when used as a croup treatment in any severity of croup when used in either isolation or placebo or when used as alternative treatment to dexamethasone.

### 3.5 Comparative Intervention

Only studies that examine prednisolone as a stand-alone treatment for croup, or compare prednisolone to dexamethasone will be used.

### 3.6 Outcomes

What is being measured is the reduction in symptoms in the children, based on the use of either drug, but only when compared to each other, *i.e.* primary outcomes are changed in clinical croup scoring from baseline to time in the future as defined by the researchers. Which drug they received, how much of it, and for how they long and which the key outcomes were measured. Also return visits and readmissions are secondary outcomes, which are measured.

### 3.7 Time Frame

2000-2016.

### 3.8 Review Questions

The questions that this systematic review seeks to answer are:

1. Is a single dose of oral prednisolone as effective as a single dose of dexamethasone at reducing croup severity score in children under 12 years?

2. What is the optimum dose of prednisolone to prevent relapsing symptoms and re-admission?

### 3.9 Search Strategy

A methodical approach based on PRISMA guidelines for undertaking reviews using electronic databases to search the literature, which was supplemented by hand searching and cross referencing. The search was based on a pre-determined series of keywords which are related as follows: Croup AND Prednisolone AND dexamethasone. Fig. (**1**) shows the combined results of the searches in a PRISMA flow diagram.

The time period is selected on the basis that around 2000 onwards, the intervention of using steroids became more widespread with some of the biggest steroid trials in mild croup being conducted in the early part of that decade.

### 3.10 Databases

The following databases were searched:

CINAHL (Cumulative Index of Nursing and Allied Health Literature);

MEDLINE EBSCO;

MEDLINE OVID;

PubMed;

ProQuest;

EMBASE;

Joanna Briggs Institute;

SUM search;

OpenGrey;

Cochrane Database of Systematic Reviews (CDSR);

Cochrane Central Register of Controlled Trials (CCRCT);

BIOSIS and Health Services/Technology Assessment (HSTAT) and

The National Research Register.

In addition, AE wrote to trial authors of studies, and the two who replied both reported that no unpublished results were available at that time.

### 3.11 Inclusion and Exclusion Criteria

**Inclusion:**

- RCTs, Case controlled studies and cohort studies.
- Studies needed to be published in English and full text available.
- Content relates directly to croup and its treatments involving prednisolone alone or in comparison to dexamethasone.
- Publication of a research study within a peer-review journal.
- Research must be related to the treatment of croup in children.
- Research must include prednisolone alone or in comparison to dexamethasone, noting that oral or intra-muscular treatments were the preferred methods of treating croup for this systematic review.

**Exclusion:**

- Studies that did not examine prednisolone or dexamethasone in relation to croup.
- Studies that used other drug therapies such as nebulised adrenaline or budesonide.
- Studies that examined rectal dosing of prednisolone.
- Studies that just examined dexamethasone.
- Studies pertaining to cases when treatment was such that children were admitted to an Intensive Therapy setting.
- Studies involving children with illnesses other than croup, for example upper respiratory tract infections, asthma, or bronchiolitis.
- Studies examining severely ill children or children with a structural abnormality of the upper respiratory tract.

### 3.12 Screening

A two-stage process was used during screening. Stage one involved screening the article and the abstract against the inclusion criteria. If there was uncertainty over the suitability of the publication, the full text of the article was assessed. Potential usefulness was identified from the abstracts by the initial search for appropriateness to the study question. Relevance was based on the review of the title and the abstract. Two studies were excluded because they were systematic reviews. Stage two involved screening of the full text of articles against the inclusion criteria. Studies examining rectal doses or the ones that examined children who were intubated were also excluded because they did not appear relevant to study or its aims, and not did not seem applicable to primary care or general practice.

### 3.13 Quality Assessment

Following the full-text selection, the studies were assessed for methodological quality. Systematic reviews rely substantially on the assessment of the methodological quality of the individual trials, and quality assessment allows means that papers can be excluded papers or weighted in the analysis phase of the review, and to determine whether research quality makes a difference to the nature of the findings [47, 48]. The quality of the randomised controlled studies and the chart reviews were assessed using the QualSyst tool for quantitative studies. The scoring system is peer-reviewed [38] and based upon established quality assessment tools for quantitative studies. To use the QualSyst assessment, each study is scored according to the degree to which they meet 14 criteria (yes = 2, partial = 1, No = 0). Items not applicable to a particular study design can be marked N/A and can be excluded from the total summary score [49].

In this systematic review, one reviewer (AE) and another author (GW) reviewed the studies independently, and both graded the quality of each paper to be included. Both authors agreed on the same quality scoring of all the papers included. All the studies selected met the minimum threshold of a summary score of 0.6. Four studies met all the criteria for inclusion.

## 4 RESULTS OF THE SYSTEMATIC REVIEW AND CRITICAL APPRAISAL

### 4.1 Heterogeneity

In the context of systematic reviews, statistical meta-analysis of findings is not always possible. Meta-analysis should only be considered when a group of studies is sufficiently homogeneous in terms of participants, interventions, and outcomes in order to provide a meaningful summary [50]. Since all of the studies included measured different outcomes, used different drug doses, and differing lengths of treatment time, it was not possible to pool the collective results and so a narrative approach was taken. This is outlined in Table **2**. Narrative synthesis aims to summarise and explain the findings of the synthesis primarily relying on the use of words and text to summarise (rather than statistics) and should encompass the analysis of the relationship within and between the studies, as well as provide an assessment of the evidence [51].

### 4.2 Randomisation and Blinding

All of the randomised controlled trials in this review reported sufficient information for them to be assessed as adequately randomised with adequate concealment of allocation and blinding of the participants and researchers [56]. Allocation bias can occur when the measured treatment effect differs from the true treatment effect because of how participants were selected into the intervention or control groups [57]. To avoid this, patients should remain unaware of which treatment is being given until the study is completed [58], and this was achieved by double blinding the participants and the researchers in all the RCTs. It must be noted that allocation concealment is completely different when blinding the former, which seeks to eliminate selection bias during the process of recruitment and randomisation, whereas blinding the latter seeks to reduce performance and ascertainment bias after randomisation [59].

There was no selection bias in the RCTs, which is concerned with systematic differences arising between the sampling population and the sample drawn. Selection bias occurs when the subjects studied are not representative of the target population about whom the conclusions are to be drawn [60]. All the children were randomly selected upon entering the trial from either the emergency department or the selected primary care offices, although they needed to have similar baseline observations and follow-up ability as this was important. Selection bias as one of the major types of bias that can impair the results of a randomised control trial but due to the nature of the design of a trial it can, and should be, avoided [61]. Randomised control trials have the unique advantage of using randomisation as a method of determining patient allocation to treatment, which eliminates selection bias if correctly executed notes that bias can cause estimates of association to be either larger or smaller than a true association [62, 63]. Also randomised control trials rest on internal validity, which is based largely on the power of randomisation, to ensure that the only difference between two treatment arms is their exposure to the treatment of interest [63].

All of the studies clearly reported the number and the ages of the children who participated in the studies as well as the inclusion/exclusion criteria. In all of the RCTs, the studies reported co-interventions. For example, use of budesonide or adrenaline, or exclusion of children due to the severity of the illness. In fact, all of the studies reported dropouts, or children who needed intubation, or those whose parents withdrew consent.

### 4.3 Sample Sizes and Attrition

New research studies should seek preliminary evidence that the intervention is likely to be beneficial (from other similar studies). Such information is needed to estimate sample sizes and justify the expense of a trial [58]. Since information is based on previous studies, a size calculation can be made based on whatever clinically meaningful difference is considered important to be detected [64].

The three trials used the intention to treat approach [52-54]. All of these studies appeared to report all the outcomes initially stated as their objectives. This means that patients were normally analysed within the group to which they were allocated, irrespective of whether they continue to experience the intended intervention or not (intention to treat analysis) [58].

The size varied in the trials but was generally small (under 200). However, to account for heterogeneity, RCTs need to be quite large to achieve statistical significance. Garbutt **et al.** [54] acknowledge recruiting issues in their trial with authors being unable to recruit their target sample of 200 patients, so much so that they remarked that there is a failure to demonstrate a significant difference between the two study drugs because of inadequate power associated with the small sample size [54]. Like Garbutt **et al.**’s trial [54], Fifoot and Ting [53] found that their trial was powered sufficiently to give primary outcome of reduction in the Westley croup score. However, they had insufficient numbers to prove that there would be no difference in representation between the groups’ secondary outcomes. Also, inflation effect can be present in small low-powered studies, which can only detect effects that happen to be large [65]. Parker **et al.** [55], only analysed data from 188 children using descriptive statistics but did not include a power calculation for their trial. Every retrospective chart review requires a statistical power analysis to determine the appropriate sample size. Calculating this appropriate sample size is a necessary component in all research proposals and is dependent on the statistical tests used in the study [66].

Without detailed information regarding the sample size calculation used when publishing papers type II statistical errors cannot be discounted [67]. Relatively large samples obtained by probability or non-probability sampling are generally used for quantitative theory-testing research designs [42] as seen in the larger studies. Researchers have an ethical responsibility to recruit an adequate size for their trials [42], but it is unethical to include more research participants than are actually needed to obtain accurate data [68]. The minimum accepted level is considered to be 80%, which means there is an eight in ten chance of detecting a difference of the specified effect size [69]. Usually, most studies accept a power of 80%. This means that it is accepted that one in five times (that is 20%) real difference will be missed. Low statistical power (because of the low sample size of studies, small effects, or both) negatively affects the likelihood that a nominally statistically significant finding can actually reflect a true effect [65]. Power calculations tell us the number of patients that are required in order to avoid a type I or a type II error [70]. Nearly all quantitative studies can be subjected to a sample size calculation [70]. The statistical power describes the probability that the study will detect and affect where there is a genuine effect to be found [71]. Low power in the absence of other biases can contribute to producing unreliable findings even when all other research practices are ideal [65]. If statistical power is positively correlated with the sample size and the larger sample size, researchers will be enabled to find smaller differences, which are statistically significant [72]. Many null studies may be underpowered to detect the desired difference due to a smaller sample size [67], in which case they will be statistically inconclusive and may make the whole protocol a failure [72]. Some studies may be over-powered (too many participants) and so it is important to achieve the correct balance [69].

Garbutt **et al.** [54] did not recruit their target sample of 200 patients and failed to demonstrate a significant difference between the two study drugs, which was due to the inadequate power associated with the small sample size. Their target sample size was 100 patients per group, based on the goal of estimating the number of children needed in each arm of the trial to achieve a 95% confidence interval (CI). Fitfoot and Ting’s (2007) sample size calculations were based on detecting a difference in the croup score baseline between the three intervention groups, and based on the sample size, the difference was calculated at 33 patients per group.

Many children suffering from croup were not approached owing to the pressure on emergency staff over a busy winter period [52]. Garbutt **et al.** [54] remarked that their results might not be generalizable to other communities as they recruited patients from just one geographic area. Additionally, they did not present or discuss how representative the patients were of all the patients with croup who were cared for at the study sites. Fifoot and Ting [53] suggest that 35.4% of eligible children were missed, again due to the high clinical activity in the emergency department. The researchers retrospectively analysed the data from these children to determine if they differed from those enrolled with baseline characteristics being similar to the enrolled group. Parents who were contacted during the 1-10 days of the trial may have been suggested to be subject to participant attrition. This can be seen in longitudinal studies, which can introduce systematic bias by favouring participants who return or take part in the follow-up, and thus increase the likelihood that those with complications will be underestimated because they did not take part [73].

Although randomised trials start as high-quality evidence, they can be down-rated if most of the relevant evidence comes from studies that suffer from a high risk of bias [74]. Acknowledging there are many types of bias, these RCTs all appeared to have inconsistencies in protocol adherence and participants dropping out. In some of the studies, researchers have accounted for this particular bias. Attrition is the loss of randomly assigned participants or participants’ data [75]. This will ultimately bias an RCT's external validity by producing a final sample that is not representative of the population sampled [76]. It was noted that there appeared to be bias in Garbutt *et al* trial [54] with flaws in design conducting analysis and reporting which could have caused the effect their invention to be overestimated [77]. Garbutt **et al.**’s trial [54] was the only trial, which reported the duration of use and self-reported adherence, without differing between groups in their trial when measuring on-going symptoms. Such attrition means that the balance in baseline characteristics for those randomised may not be maintained in the subsample that has the outcome data [78]. However, the main evaluative strength of RCTs is that each group should be generally balanced in all characteristics, with any imbalance occurring by chance [79]. Conducting RCTs in paediatric research is challenging where recruiting more children than necessary risks unnecessary overexposure of children to inferior treatment, whereas underestimating the size will produce inconclusive results [64].

### 4.4 Heterogeneity of Outcome Measures

All these randomised studies specifically outlined their intent to report on whether prednisolone was as effective as dexamethasone or as in Parker **et al.**’s trial [55], how long stridor lasted for after the administration of prednisolone. The trials all measured different outcomes although the methodology was the same in the randomised double-blinded control trials and were all [52-55] explicit about their intention to study children of specific age groups and by specific methods of data collection. Although the studies were conducted in different areas, only Garbutt **et al.**’s [54] trial was based around primary care setting, whereas the others were based in Emergency Departments. All three [52-54] performed randomised double-blinded comparison trials of prednisolone *versus* dexamethasone, whereas Parker **et al.** [55] carried out a retrospective chart review questioning how long stridor at rest persists in croup after the administration of oral prednisolone.

There have been great improvements in patient’s clinical indices due to introduction of croup scoring systems [80]. A variety of scoring systems were used in these studies: the Westley croup Score [52, 55], the Taussig Croup Score [53], the Geelhoed Score [55] and the Telephone Outpatient Score (TOP) [54]. Westley Croup score is the most widely used and its validity and reliability have been well demonstrated [27]. Such heterogeneity in outcomes measures makes analysing their results problematic and statistical meta-analysis impossible.

Only three of the scoring systems used in these studies have been shown to have reliability in independent studies. The Westley Croup Score [81] has been evaluated its use with respect to inter-rater reliability, construct validity, and responsiveness to change [5] and is the only method that has undergone validation and reliability testing and has shown to be sensitive to important changes in a patient’s clinical status [14]. It performed well in all areas of assessment and inter-rater reliability between three research assistants was assessed prospectively. The weighted kappa was 0.90 for the total croup score, 0.47 for air entry, 0.93 for stridor and 0.87 for retractions [5]. Kappa was introduced as a measure of agreement in form of a test of inter-rater reliability, which adjusts the observed proportional agreement to take into account the amount of agreement which would be expected by chance, and represents the extent to which the data collected in the study are correct representations of the variables measured [82]. The Geelhoed score has demonstrated inter-rater reliability, with a weighted kappa of above 0.85 in two independent studies, indicating reasonable inter-observer agreement [83]. The Taussig score, which was used by Fifoot and Ting [53], does not appear to have been tested for validity and reliability with children experiencing croup [84] whilst the TOP score is a brief telephone outpatient scoring system that assess the presence of stridor and barky cough by asking parents about the child’s symptoms in the last 24 hours and has undergone limited testing for validity and reliability [85]. Croup scores themselves at entry point to trials are subject to both inter- and intra-observer variation because not all the nurses scoring the children are the same or it may be unlikely that the same nurse scores the same child later, meaning that the formal establishment of inter-rater reliability is important, particularly if it has not been demonstrated.

### 4.5 Children’s Ages

The ages of the children in the studies varied. It is known that croup typically affects younger children, mostly under the age of six years [5], although NICE [1] suggest that children as young as three months of age, adolescents, and very rarely, even adults can be affected with croup. Croup diagnosed before the age of six months is uncommon and argues that a child of less than six months with acute stridor should not be considered to have croup since croup is rare in young babies [86]; even so two papers did enrol children from 3 months upwards [52, 55].

### 4.6 Medication Dosages, Lengths of Time of Administration and Responses to Treatment

Further analysis of the trials showed that they measured different ranges of drugs for different lengths of time. Sparrow & Geelhoed [52] examined a single dose of prednisolone *versus* a single dose of dexamethasone. Garbutt **et al.** [54] measured three days prednisolone *versus* one day of dexamethasone (and two days placebo) Fifoot and Ting [53] administered a single oral dose of either prednisolone at 1 mg/kg, and dexamethasone at 0.15 mg/kg as well as 0.6 mg/kg in this three arm trial. Parker **et al.** [55] calculated how long stridor persisted after a single dose of prednisolone.

Garbutt *et al*’s [54] trial is the only trial of its kind, which compared multiple doses of prednisolone with a single dose of dexamethasone, something that had not been evaluated before. Garbutt **et al.** [54] suggest that evidence to support the treatment of croup with a one-off dose of prednisolone is scant but it offers a convenient and familiar treatment for the primary care management of croup, as it is commonly used for in-office treatment of asthma exacerbations. Although prednisolone has not been widely studied there is no reason to suppose it would be less effective [3].

Further differences are noted in the information and outcomes that the researchers were interested in. Parker **et al.** [55] collected patient demographics, croup scores on presentation to the emergency department, and the duration of strider at rest (SAR), which was taken to be the time from administration of prednisolone to the first clear documentation that SAR has ceased approximated to the nearest half hour. This type of retrospective study relies entirely on the completeness and accuracy of the data from the children’s medical records [87].

Sparrow and Geelhoed’s study participants [52] were also observed at 30 minutes after administration and then hourly until the four-hour mark. In comparison, Garbutt **et al.**’s [54], main outcomes were unscheduled representations to medical care as determined by telephone follow-ups at 7-10 days. Other secondary outcome measures were adrenaline (epinephrine) use, croup score, and the time spent in the emergency department. Fifoot and Ting’s [53] trial’s primary outcomes were the severity and rate of reduction in the Westley croup score, rate of return for medical care with on-going croup, and further treatment with steroids in the week following the initial presentation. Their secondary outcome measures were the proportion of subjects requiring admission or salvage therapy, such as nebulised adrenaline, during the initial presentation. Sparrow and Geelhoed’s [52] trial had similar follow-ups, although in this trial the primary outcomes were different in that the researchers wanted to know if the children who received prednisolone were more likely to represent for further medical care. Their secondary outcome measures were similar to that of Fifoot and Ting’s [53] in that they included length of time spent in the emergency department, duration of croup symptoms (again reported by parents), and use of nebulised adrenaline.

In the studies that looked at longitudinal data to report ‘on-going’ symptoms and need for further treatment for example, as in Fifoot and Ting’s study [53], it was suggest that the researchers’ methods did not distinguish between unscheduled re-attendances and planned reviews [20] which made analysis difficult. Despite this, the two studies [52, 53] had good validity with appropriate randomisation, double blinding, and over 85% follow up with similar baseline characteristics [20]. Parker **et al.** [55] used a mixture of patient demographics, which included the Westley croup score and the Geelhoed scoring on presentation to the Emergency department. Fifoot and Ting [53] used modified Taussig score to assess croup severity. In the Garbutt **et al.** [54] trial, the researchers use the Westley croup scoring, which was assessed at the child’s presentation. Sparrow and Geelhoed’s [52] study participants had mild to moderate croup as defined by clinical symptoms and Taussig croup score.

Parker **et al.** [55] noted that they found it difficult to discriminate between mild, moderate, or severe retraction from the notes, thus leading to possible discrepancies in the croup scoring of these children. Inter-rater reliability in this case would be described as the extent to which two or more people would score or rate things [45]. Parker **et al.**’s [55] retrospective data on croup treatments is generally considered inferior to prospective designs, and which has been recorded for reasons other than research [87]. Another other advantages of conducting chart reviews is its relatively inexpensive ability to research readily accessible data [66].

A potential problem with data collection through chart reviews in case of Parker **et al.**’s study [55] was that the researchers collected data (from a busy children’s ward) that may have had the potential for errors and inaccurate recording of symptoms. Intra-observer reliability of the croup scores by clinicians in the Parker **et al.** trial [55] was re-assessed by a blinded re-calculation of the scores from 10.6% of the original records selected at random from the initial data collection using two researchers. In each group, the extent to which intra-observation agreement was assessed using a weighted Cohen K score, which in this case was moderate. Intra-observation agreement in the busy practice setting of a paediatric emergency department showed the existence of substantial inter-observer variability among health care providers in the measurement of respiratory signs associated with croup in young children [88, 89].

Parker **et al.** [55] recorded measurements of prednisolone’s effectiveness at a thirty-minute mark and thereafter used this retrospective data to analyse the duration of stridor after administration of steroids as determined by nursing observations. Data collected may have been inaccurate due to the busy state of the ward and subject to recall bias, with the timings rounded up to the nearest 30 minutes. This was a surprising weakness in this study given that the objective was to determine the specific duration of stridor. However, acknowledging that the trial was conducted in a busy emergency department, 10-15 minute observations may not have been feasible or possible. The time noted for the cessation of stridor at rest (SAR) relied heavily on researcher’s finding the comment ‘nil stridor at rest’, usually found in the nursing records. The nursing notes may have been completed sometime after the actual disappearance of the SAR, and this may have under or overestimated the duration of the symptoms. Results showed that SAR at rest was well documented in the nursing notes. However, it is suggested that overall figures for calculating stridor are suggested to be too open to inter-rater variability to be able to accurately describe the severity in each group, *i.e.* the nurses graded the child’s symptoms differently.

Other examples of possible inter-rater reliability and intra-observer reliability issues in the trials were found in all three of the randomised control trials due to which the studies had limitations because the subjective information from parents/guardians in the follow-up telephone reviews would have relied entirely on the parents accurately describing their child’s symptoms. Although, Sparrow and Geelhoed’s [52] main outcomes’ measure was determined by unscheduled re-presentation to medical care, as determined by a telephonic follow-up at 7-10 days, which was less inclined to erroneous data from parents.

Fifoot and Ting [53] interviewed the parents of 86 patients (87%) in 1 week, asking them to recall symptoms and further needs for care. Garbutt **et al.** [54] also collected similar outcomes with telephone interviews on days 1, 2, 3, 4, and 11. Intra-observer reliability issues that were noted by this particular approach were the potential issues of recall bias as the information might have been extremely subjective. Information gathered depended entirely on memory, which can often be imperfect and thereby unreliable, as in the case of people who have difficulty remembering or accurately retrieving incidents that happened in the past, leading to poor versions of the original percept. Recall bias is a classic form of information bias and represents a major threat to the internal validity of studies that use self-reported data [90].

When considering the factors that might explain any differences in the direction and size of effect seen across the included studies, further analysis of the studies showed that a proportion of the children treated by Garbutt **et al.** [54] (which evaluated three days of treatment with prednisolone (2/mg/kg) *versus* one dose of dexamethasone for croup) needed treatment with further steroids at with the same frequency, regardless of the initial treatments assignment. This concluded that prednisolone at initial presentation and at one and four hours is as effective as dexamethasone. Garbutt **et al.** [54] remark that they did not demonstrate whether prednisolone prevents representation after the initial presentation, thus overall supporting the use of prednisolone for croup. This was disappointing as their three-day trial of prednisolone was the first of its kind. In contrast, the Sparrow and Geehoeld’s [52] trial reported on unscheduled representation rates and found that 7% of the dexamethasone group returned for care, whereas 29% of the prednisolone group showed that a single dose of prednisolone does not work as well as a single dose of dexamethasone for children with mild or moderate croup. The absolute difference of 22% between the groups had 95% confidence intervals between 8% and 35%, which was well outside the authors’ definition of equivalence [52]. Further analysis showed that dexamethasone worked significantly better than prednisolone (P < 0.01) on this measure [52]. Based on this study, however, the authors recommend that if prednisolone is used, a two-day course of 1-2 mg/kg is probably justified.

In comparison, Fifoot and Ting [53] found no difference between the three groups of prednisolone when compared to 0.5 mg dexamethasone or 0.6mg dexamethasone, reporting that there were no significant differences in primary or secondary outcome measures. However, Johnson [27] while reporting on the Fifoot and Ting study [53] proposed different findings as 13% of the dexamethasone 0.15 mg group re-attended a week later, *versus* 11% of the dexamethasone 0.6% group *versus* prednisolone 1mg / kg, which equals to 17%. Furthermore, he criticises the trials for not reporting on the significance of each dexamethasone group alone *versus* prednisone alone. Fifoot & Ting [53] disagree, suggesting that both prednisolone at 1 mg/kg and a low dose of dexamethasone (0.15 mg/kg) were found not to differ in efficacy. Children randomised to dexamethasone were significantly less likely to have a return visit/re-admission than those randomised to prednisolone (RR 0.3, 95% CI 0.2 to 0.6; I^2^ statistic 0%) [14], which contradicts the author’s own findings of no significant differences in primary or secondary outcome measures. Johnson (2008) was also critical of both trials [52, 53] as their GRADE analysis highlighted they had quality points being deducted for incomplete reporting of results, and not carrying out between-group assessments and their inconsistency in reporting of results.

Parker **et al.**’s [55] chart review found that symptoms of croup recovered quickly after administration of prednisolone but did not report on the need for on-going care after the initial treatments as the other trials did. Further analysis of the chart review data showed statistical analysis of the children’s croup scores, demographics, and cessation of SAR, concluding that the median time for SAR was 6.5 hours in children who had been given prednisolone.

### 4.7 External Validity

It is likely that the applicability evaluated in the included studies is representative of, or can be reproduced in, usual clinical care. This is important and can be seen as a consideration in all the trials presented with the extent to which external validity is representative of the study population and thus likely to be representative of the general population [91]. Appraising the applicability of the results of a study is intertwined with the quality of reporting, *i.e.* the extent to which an article provides information about the patients, the intervention, and the context of care which is good in all of the studies. Often there are concerns about generalizability of trials not in secondary or tertiary care when practiced in primary care. However, we believe that hospital studies would not be any less representative of the types of children seen or whether the outcomes would be any different from those observed in primary care settings [92], except that the children may be sicker. External validity in any of the studies presented is not a concern as the randomly selected results of the chart review are selected from one geographical area and it is suggested the RCTs are very likely to representative of the general population who use the treatment [93].

## 5 SUMMARY OF REVIEW FINDINGS

Four studies [52-55] met the inclusion criteria, but they were too heterogeneous to combine in statistical meta-analysis. Narrative synthesis was undertaken, which suggests that although prednisolone appears as effective when first given, it is less so for preventing re-presentation. However, because trial sample sizes were small, outcomes measures and croup scoring methods so different, firm conclusions are difficult, but it appears as a second dose of prednisolone the following day may be useful. We are unable to state the optimum dosages required.

## 6 DISCUSSION

Unfortunately, the four studies included were too heterogeneous for statistical meta-analysis to take place. Combining studies that are not similar can cause significant inaccuracies in summary effects as well as associated conclusions, thus misleading decision makers and others [94] and so by not attempting to combine them we have avoided made this mistake. Variability in the intervention effects being evaluated in the different studies is known as statistical heterogeneity, which is a consequence of clinical or methodological diversity [50]. While exploring relationships within the data from this systematic review, there was some difficulty in organising the findings from the studies and describing patterns across the studies, in terms of the direction of effect and of the size of effects because the studies measured different outcomes and used differing methods. Measuring outcomes is suggested to be a key factor in analysis of the trials [25, 28], but there was too much inconsistency in outcomes assessment because of the different croup scoring scales or worse, no croup scoring, within the studies [52-55]. Where secondary outcomes such as on-going symptoms are vague, this may be due to poor inter-rater reliability [95].

## 6.1 Clinical Effectiveness

The relatively small sample sizes, heterogeneity of outcomes and effects, and other limitations noted above make this systemic review limited as a means of indicating clinical effectiveness’ assessing optimum dosages and rates of re-presentation, which were our original aims. Although three of the studies are RCTs, which might indicate that this body of evidence sits towards the top of accepted hierarchies of evidence, this systematic review would be rated weak as a guide for clinical practice [45].

## 6.2 Grey Literature and Eliminating Bias

A significant amount of time was devoted to identifying grey literature. Authors of the studies included in the literature review were written to. A review of the materials found in the reference lists of included studies was also conducted by looking through university theses as well as by hand searching of articles, conferences, reports, and opinion pieces. It is suggested that grey literature is particularly difficult to identify and retrieve with some databases, such as the National Research Register, listing unpublished work [96]. By including unpublished evidence, it may be possible to minimise the impact of the bias towards publishing only positive results in the literature [97]. The validity of a systematic review is highly dependent on the results of the underlying data, and the inclusion of grey literature may help to overcome some of the problems of publication bias, which can arise due to the selective availability of data [98]. The most consistent difference between published and grey literature is that published research is more likely to contain results that are statistically significant and can contain effect size estimates that are about one-third larger than those of unpublished studies [99]. Although time-consuming and costly, literature searches which cover the grey literature in all relevant languages and databases, are normally recommended to prevent reporting biases, but this was not possible in the systematic review as resources did not allow employment of speakers of languages other than English to conduct searchers. It is not clear how much this systematic review would be affected by a lack of grey literature in non-English languages, which is therefore acknowledged as a potential sources of bias. We were not able to construct funnel plots to assess publication bias due to the small number of studies and their relative heterogeneity, which is a further limitation of this systematic review.

## 6.3 Quality of Included Studies

Quality assessment is an integral part of a systematic review because if the results of individual studies are biased, and these are synthesised without any consideration of quality, then the results of the review will also be biased, and the quality of the evidence and conclusions generated by a systematic review depends on the quality of the primary studies that make up the review [100, 101]. We have included details of our quality assessment processes, involving independent assessment by two researchers (AE and GW), and this process gave consideration to the methodological quality of studies, including allocation concealment, randomisation and comparability of the group’s baseline characteristics, treatment adherence and participation and was based on established processes [38]. Trials with low methodological quality and small sample sizes can result in misinterpretation of RCT’s overestimated differences in effectiveness or undetected, smaller, but statistically significant differences, and we have acknowledged these issues in the detailed critical analysis above. None of the included studies were scored less than 0.6, which is an acceptable cut off point for inclusion [38]. Higher quality scores should indicate studies with a better methodological quality [47] but methodological quality is likely to remain relatively subjective, as has been the case in this systematic review.

## CONCLUSIONS AND RECOMMENDATIONS

Overall, the evidence from this systematic review seems to suggest that although prednisolone appears as effective when first given, it is not as good at preventing re-presentation. There continues to be a paucity in the existing literature as to whether prednisolone is as effective as dexamethasone. Fifoot and Ting [53] agree and also conclude that dexamethasone and prednisone are equally effective when initially given and also are equally effective at preventing readmission, finding no difference between oral dexamethasone and oral prednisolone in case of croup score at four hours or in terms of rate of return for medical care [53], although they concluded that at that time there was not enough evidence either way to conclusively establish that prednisolone is as good, but early on in treatment, *i.e.* in the first 6 hours, the ‘Stridor at rest’ resolved promptly after prednisolone and was as effective as dexamethasone [55]. Even so, more investigation is needed to evaluate the comparable efficacy. It is likely that a large proportion of the children, who would have been previously admitted to the hospital, would have been treated and discharged from the emergency department after having prednisolone [55].

The optimum dose of prednisone to prevent relapsing symptoms and readmission is still unclear and requires further research. A comparable efficacy between a single dose of dexamethasone and single oral dose of prednisolone (1 mg/kg) for mild to moderate croup has been suggested elsewhere [102], but Garbutt **et al.** [54] concluded that when a three-day course of prednisolone is administered, the researchers found this approach to be equivalent to a single oral dose of dexamethasone (0.6/mg/kg), once again highlighting that prednisolone and dexamethasone seem equally effective when first given but relapse and re-attendance to medical care is more common with prednisolone in children with mild or moderate croup, although again there is disagreement about this elsewhere [103]. There is an absence of evaluative research on prednisolone compared to dexamethasone in respect to repeated doses but also a view that it would not be palatable [14, 24]. No other studies as yet have compared the effectiveness of dexamethasone and multiple doses of prednisolone for the treatment of croup in the community setting, but this systematic review indicates that clinicians might be able to feel confident giving a repeat dose of prednisolone the following day, should the child still have stridor or other residual symptoms such as a ‘barking cough’, particularly if they are followed up by telephone, as did Garbutt *et al* [54]. Prednisolone is widely regarded as an alternative to dexamethasone for croup [29] but there remain questions over the evidence base for dosages: as well as the regimens listed above, UK guidelines suggest a repeat dose of prednisone the following day, but only if there are residual symptoms [14]. The British National Formulary (BNF) [104] only recommend 0.15/mg/kg of dexamethasone and do not recommend prednisolone, unless the child has severe croup and is being admitted to hospital where they recommend a single dose of 0.15mg / kg dexamethasone and administer 1-2 mg/ kg of prednisolone when the former is not available. Oral prednisolone at 1-2 mg/kilo is an alternative if dexamethasone is not available but no further advice on repeat dosing is given[1].

There may be a possible economic benefit to using prednisolone, as prednisone is significantly cheaper than dexamethasone and seems to be more widely available. Besides, soluble prednisolone is probably more widely available and cheaper than liquid dexamethasone. For treating a 12 kg child, the prednisolone dose would cost £0.75 compared to £2.50 for 0.15 mg/kg of dexamethasone [104]. Even if a 2-day course of prednisolone is given, it still results in a 40% cost saving [20], therefore a recommendation of this systematic review is that when further studies are undertaken, cost-benefit analysis is part of the study design.

More research is needed which examines direct comparison trials of both drugs with sufficient numbers and length of trials with appropriate reliable outcomes of primary care. In general, primary care manages the vast majority of children with mild croup, with only one RCT evaluating interventions in true primary care settings [54]. Large trials are needed that examine single or multi dose of prednisolone at the kind of numbers with which Bjornson **et al.** [7] used: this would give clinicians more robust evidence that prednisolone is as effective as dexamethasone in treating croup. Trials testing a longer course of prednisolone should be done in places where dexamethasone elixir is hard to come by [35]. There is an argument that those conducting further research studies should standardise their outcome measures, as a minimum there needs to be some consensus as to whether the Westley or Geelhoed scoring systems, or indeed both, should be utilised.

This systematic review highlights the most available and up to date research and its methods in this area and on a practical level, we believe, shows that prednisolone is as effective when first given and that two doses are far more likely to control symptoms at that level. It is very likely that small, short doses of prednisolone are safe for children, may be well tolerated and improve symptoms quickly. Given the data reviewed, prednisolone appears to be an appropriate choice in the treatment of mild to moderate croup where dexamethasone is not available. It is no longer reasonable to conclude that the use of corticosteroids should be reserved for individuals who are hospitalised with moderate to severe croup [3], with prednisolone offering a convenient and familiar treatment for the primary care management of croup [54].

## Figures and Tables

**Fig. (1):**
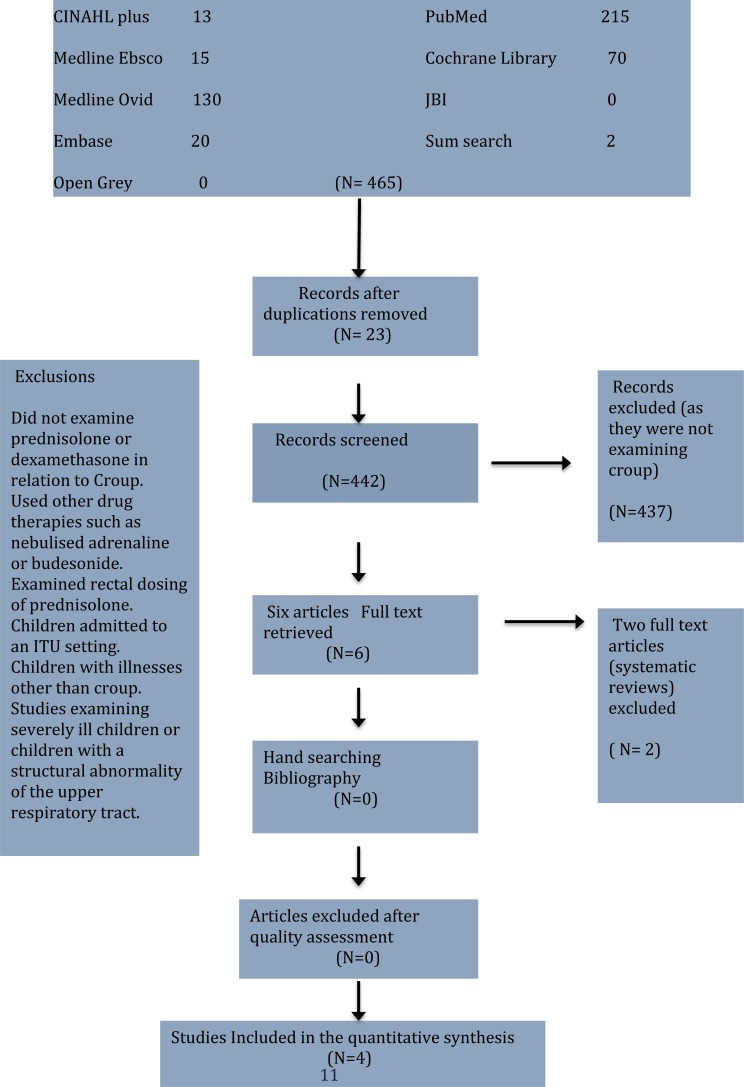
Combined results of the searches in a PRISMA flow diagram.

**Table 1:** PICO employed in this systematic review.

**P**	**P**atient, Population, or Problem	How would I describe a group of patients similar to mine? **Children with croup < 12 years of age.**
**I**	**I**ntervention, Prognostic Factor, or Exposure	Which main intervention, prognostic factor, or exposure am I considering? **Prednisolone or dexamethasone**
**C**	**C**omparison or Intervention (if appropriate)	What is the main alternative to compare with the intervention? **Studies that examine prednisolone usage or compare prednisolone with dexamethasone**
**O**	**O**utcome you would like to measure or achieve	What can I hope to accomplish, measure, improve, or affect? **Measured symptom reduction, dosages and re-presentation rates.**

**Table 2:** Relative heterogeneity of the included studies.

Author	Patient Group and Age	Study Design	Outcomes	Key Results	Study Weaknesses
[52] Sparrow and Geelhoed, 2006.	Children aged 3 months to 142 months old presenting to a single emergency department with mild to moderate croup.	Randomization and blinding Double blind, randomised equivalence study. Sample size and attrition 133. No attrition Medication dosages Patients received a single dose of 0.15 mg/kg dexamethasone 0.6 dexamethasone or 1 mg/kg prednisolone. Lengths of time of administration Single dose of prednisolone, 1 mg/kg, matched for potency with a single dose of dexamethasone in children with mild to moderate croup.	Primary outcome was the magnitude and rate of reduction in Westley croup score. Clinical observations at 30 minutes after administration of steroid; hourly for the next four hours and four hourly thereafter until discharge. Criteria for discharge home were minimal stridor or chest wall retractions—that is, a croup score of 1 or 0. Re-attendance at medical care within 7-10 days.	Responses to treatment: Five out of 68 (7%) children who had received dexamethasone returned for medical care *versus* 19/65 (29%) children who had received prednisolone. No significant difference between the three groups in magnitude or rate of Westley score reduction.	Included patients up to the age of 12 years, although very uncommon for older children to be affected. Exclusion criteria included prior administration of steroids, non-English speakers and no access to telephone. Small numbers.
[53] Fifoot and Ting, 2007	Children 6 months to 6 years presenting to a single emergency department with mild or moderate croup	Randomization and blinding Double blind, randomised trial. Sample size and attrition 99 children with 86 patients followed up by telephone (87%). Medication dosages Patients were randomised to receive 1 mg/kg prednisolone, 0.15 mg/kg dexamethasone or 0.6 mg/kg dexamethasone. Lengths of time of administration Single dose.	Primary outcome measures were the magnitude and rate of reduction in Westley croup score, rate of return for medical care with ongoing croup, and further treatment with steroids in the week following index presentation. Secondary outcome measures were the proportion of subjects requiring admission or salvage therapy, such as nebulized adrenaline, during index presentation. Follow-up by telephone interview at 7 days, Taussig score.	Responses to treatment No significant difference in admission rates, duration of symptoms or attendances. No significant difference between treatment groups.	Small sample size. Large number of eligible patients not recruited. Primary outcome over short time period.
[54] Garbutt *et al*, 2013	Children aged 1-8 years presenting to primary care offices with mild or moderate croup in the USA.	Randomization and blinding Double blinded randomised comparison trial Sample size and attrition Eighty-seven children randomised with 98% follow up at 11 days. Medication dosages and lengths of time of administration Prednisolone 2mg/ kg per day for 3 days *versus* one dose of 0.6mg dexamethasone and two placebo doses	Additional health care within 11 days of randomization assessed by self-report Telephone Out Patient score. Secondary outcomes included: duration of croup symptoms; disturbed sleep; parental stress; adverse events including sleep problems, mood changes, headache or dizziness, nausea, stomach pain, and secondary infections.	No difference in outcomes of either group for child or parent.	Small numbers. People with no telephone and lacking English spoken language were excluded.
[55] Parker *et al*, 2004	Children aged 4 months to 11 years, median age 2 years presenting in an emergency department with mild or moderate croup	Retrospective chart review. Randomization and blinding Not undertaken Sample size and attrition 188 eligible for analysis from 814 patients coded as croup. Medication dosages 1mg/kg prednisolone. Lengths of time of administration Not specified but implicitly single dose only	How long stridor at rest persisted after administration of prednisolone 1mg /kg. Westley and Geelhoed croup scores. To determine whether children with mild croup had a more rapid resolution of stridor at rest.	Average length of time from SAR to cessation was 6.5 hours. Children with mild croup improved quicker although this is reported as not clinically significant.	Retrospective chart reviews rely on completeness of contemporaneous data entry. Inter rater reliability reported as moderate. Lack of power calculation

## LIST OF ABBREVIATIONS

BNF: The British National Formulary
NICE: National Institute for Health and Care Excellence
PRISMA: Preferred Reporting Items for Systematic Reviews and Meta-Analyses
RCT: Randomised Controlled Trial
SAR: Stridor at Rest
TOP: Telephone Outpatient Score
